# Intraperitoneal Isoastragaloside II Attenuated Western Diet-Induced Atherosclerosis in Male ApoE^-/-^ Mice with Lipid-Lowering and Anti-Inflammatory Effects

**DOI:** 10.3390/nu18193137

**Published:** 2026-09-23

**Authors:** Jian Ai, Xin Tang, Haorui Ma, Deshan Ning, Yong Zhou, Bingyong Mao, Qiuxiang Zhang, Jianxin Zhao, Wei Chen, Shumao Cui

**Affiliations:** 1 State Key Laboratory of Food Science and Resources, Jiangnan University, Wuxi 214122, China; 2 School of Food Science and Technology, Jiangnan University, Wuxi 214122, China; 3 Infinitus (China) Company Ltd., Guangzhou 510405, China; 4 MOE Medical Basic Research Innovation Center for Gut Microbiota and Chronic Diseases, School of Medicine, Jiangnan University, Wuxi 214122, China; 5 National Engineering Research Center for Functional Food, Jiangnan University, Wuxi 214122, China; 6 Wuxi Institute for Specialized Nutrition and Health Co., Ltd., Wuxi 214122, China

**Keywords:** Isoastragaloside II, atherosclerosis, foam cells, lipid-lowering, inflammation

## Abstract

**Background**: Atherosclerosis is driven by lipid accumulation, vascular inflammation, and macrophage foam cell formation. Nutritional therapy, employing natural products with bioactive compounds, is a potential avenue for treating atherosclerosis. Isoastragaloside II (Iso-AS-II) is a bioactive saponin isolated from the medicinal and edible plant *Astragalus membranaceus* (Huangqi), but its role in atherosclerosis has not been investigated. **Methods**: In this study, we investigated the effects of Iso-AS-II (i.p., 5 mg/kg) in male ApoE^-/-^ mice fed a Western diet (WD) and identified potential mechanisms through network pharmacology and transcriptomic analyses. In parallel, oxidized low-density lipoprotein (ox-LDL)-induced RAW 264.7 foam cells were used to evaluate the efficacy of Iso-AS-II in reducing foam cell accumulation and inflammation. **Results**: Iso-AS-II reduced aortic lesion area, plaque macrophage infiltration, and smooth muscle cell content, and lowered serum lipids (TC, TG and LDL-C) and inflammatory cytokines (TNF-α, IL-6, MCP-1, M-CSF and IL-12). Network pharmacology and transcriptomics suggested modulation of lipid metabolism and inflammatory pathways. In macrophages, Iso-AS-II suppressed foam cell formation, accompanied by downregulation of *Cd36* and *Msr1* and upregulation of *Abca1* and *Abcg1,* and exerted anti-inflammatory effects by reducing IL-1β, IL-6, and TNF-α levels. **Conclusions**: This exploratory study provides the first evidence that Iso-AS-II reduces atherosclerotic burden in WD-fed ApoE^-/-^ mice, and suggests that these effects involve improved systemic lipid homeostasis, suppression of inflammation, and inhibition of macrophage foam-cell formation, supporting its potential as a natural anti-atherosclerotic agent.

## 1. Introduction

Cardiovascular diseases (CVDs) are the leading causes of mortality and are among the foremost causes of disability globally [1]. Atherosclerosis has been recognized as a major risk factor for CVDs. Atherosclerosis is a chronic inflammatory disease of the arterial wall and remains the leading cause of myocardial infarction, stroke, and peripheral artery disease worldwide [2]. Atherosclerosis is pathologically characterized by the formation of plaques containing lipids, connective tissue, and immune cells within the arterial wall, and its mechanisms involve dyslipidemia, endothelial injury, inflammation, and other contributing factors [3]. Although statins and other lipid-lowering agents reduce cardiovascular events, they do not fully eliminate “residual cholesterol risk” and “residual inflammatory risk”, and long-term therapy can be limited by incomplete lipid lowering or intolerance [4]. These therapeutic gaps have encouraged the search for new therapeutic agents with simultaneous lipid-modulating and anti-inflammatory activities. Nutraceuticals are natural nutritional compounds that are beneficial for the prevention or treatment of disease and, therefore, are a possible therapeutic avenue for the treatment of atherosclerosis [5]. In particular, plant-derived natural products have become a highly attractive source for anti-atherosclerotic drug discovery, owing to their safety, mild adverse effects, and multi-target synergy [6,7].

*Astragalus membranaceus* (Huangqi) is a medicinal and edible plant that has been widely used in traditional Chinese medicine and as a dietary supplement for centuries. Its major saponin constituents, astragalosides, are cycloartane-type triterpenoids with anti-inflammatory, immunomodulatory, and cardioprotective activities [8]. Although astragaloside IV has been extensively investigated, the biological activities of other astragaloside congeners remain largely unexplored. Isoastragaloside II (Iso-AS-II) is a bioactive cycloartane-type saponin isolated from *A. membranaceus*. Structurally, Iso-AS-II differs from astragaloside IV primarily by the presence of an acetyl group, suggesting that it may possess similar or potentially distinct biological activities. Iso-AS-II has been reported to inhibit lipopolysaccharide (LPS)-induced nitric oxide production in RAW 264.7 macrophages [9], and to modulate PPAR-α/FXR signaling and bile acid metabolism, thereby ameliorating cholestatic liver diseases [10]. Whether Iso-AS-II influences atherosclerosis, and if so through which mechanisms, remains unknown. In this study, we therefore evaluated the effects of Iso-AS-II on atherosclerosis in Western diet (WD)-fed ApoE^-/-^ mice and explored its potential mechanisms of action.

## 2. Materials and Methods

### 2.1. Animal Experimental Design

Specific pathogen-free (SPF) male ApoE^-/-^ mice (6 weeks old, 20 ± 2 g) were purchased from GemPharmatech Co., Ltd. (Nanjing, China). The animals were housed in the barrier facility of the Laboratory Animal Center at Jiangnan University under standard conditions (12 h light/dark cycle, 22 ± 2 °C, 50–60% humidity) with free access to food and water. All animal procedures were approved by the Institutional Animal Care and Use Committee of Jiangnan University (approval No. JN.No20241030t1100214[553], approval date: 30 October 2024) and performed in accordance with the institutional guidelines for the care and use of laboratory animals.

After 1 week of acclimatization, the ApoE^-/-^ mice were randomly assigned to three groups (*n* = 8 per group, four per cage, totaling 24 mice): normal chow diet (NCD), Western diet (WD, or high-fat and high-cholesterol diet), and WD plus Iso-AS-II. The NCD group received a standard diet (#XT102C), while the WD and Iso-AS-II groups were fed a high-fat, high-cholesterol diet (#XT108C). All diets were purchased from Jiangsu Xietong Pharmaceutical Bio-engineering Co., Ltd. (Nanjing, China). The diet composition is presented in Supplementary Table S1. Mice in the Iso-AS-II group received Iso-AS-II (≥99% purity, BioBioPha, Kunming, China) at 5 mg/kg (intraperitoneal injection, i.p.), dissolved in a vehicle containing 5% DMSO, 5% Tween 80, and 90% saline. The dose of Iso-AS-II (5 mg/kg, i.p.) was selected based on a previous study in which 5 mg/kg (corresponding to an average concentration within the mouse body of approximately 6.0 µM) astragaloside IV was used as an effective intraperitoneal dose in mice [11]. The NCD and WD groups received an equivalent volume of vehicle. Iso-AS-II was administered by i.p. five times per week for 12 weeks at approximately 0.2 mL per injection.

Body weight was recorded weekly, and fresh diet was provided every 3 days. At the end of the experiment, all mice were euthanized by cervical dislocation after being anesthetized with isoflurane, and blood was collected from the abdominal aorta. Blood samples were allowed to clot at room temperature for 30 min and then centrifuged at 3000 rpm for 15 min at 4 °C. The serum was collected and stored at −80 °C for further analysis. The aorta was then collected, and a portion of the aorta was fixed in tissue fixative solution (4% paraformaldehyde) for histological analysis, while the remainder was stored at −80 °C.

### 2.2. Serum Biochemical Analysis

Serum levels of total cholesterol (TC), triglycerides (TG), low-density lipoprotein cholesterol (LDL-C), high-density lipoprotein cholesterol (HDL-C), alanine aminotransferase (ALT) and aspartate aminotransferase (AST) were measured using a Mindray BS-480 automated biochemical analyzer (Mindray, Shenzhen, China).

### 2.3. Luminex Analysis

Thawed mouse serum samples were analyzed using a Luminex multiplex cytokine assay (LabEx, Shanghai, China) for the following markers: TNF-α, MCP-1, IL-1β, IL-4, IL-6, IL-10, IL-12, and M-CSF.

### 2.4. Oil Red O Staining of the Aorta

Aortic tissues fixed for over 24 h were washed twice with PBS, opened longitudinally, rinsed with distilled water, immersed in 60% isopropanol for 3 s, and stained with Oil Red O working solution at 37 °C in the dark for 1–2 h. After differentiation in 60% isopropanol, staining was stopped by a water rinse. Aortas were blotted, spread flat on a glass slide over a black background, and photographed.

### 2.5. Histopathology and Immunofluorescence Analysis

Atherosclerotic lesions in the aortic root were evaluated by H&E staining and Masson staining, and immunofluorescence staining for F4/80 (macrophage marker) and α-SMA (smooth muscle cell marker). Tissue processing, sectioning, and staining were performed by Servicebio Technology Co., Ltd. (Wuhan, China). For immunofluorescence, sections were incubated with primary antibodies overnight at 4 °C, followed by secondary antibody incubation and tyramide signal amplification (TSA). Nuclei were counterstained with DAPI. Images were acquired using a digital slide scanner and analyzed with CaseViewer (v2.4.0), ImageJ (v1.8.0), and Image-Pro Plus (v6.0) software.

### 2.6. Transcriptomic Analysis

RNA sequencing and data analysis were performed according to the methods described in previous studies [12]. Briefly, total RNA (*n* = 4 per group) was extracted from aortic tissues using TRIzol reagent. RNA concentration and purity were assessed with a NanoDrop 2000 spectrophotometer (Thermo Fisher Scientific, San Jose, CA, USA), and RNA integrity was evaluated by agarose gel electrophoresis and further verified using an Agilent 5300 Fragment Analyzer (Agilent, Santa Clara, CA, USA). Samples with a total RNA amount > 0.5 μg, concentration ≥ 20 ng/μL, RNA quality number > 4.5, and no detectable contamination were used for library preparation. Library construction, sequencing, and bioinformatic analysis were performed by Majorbio Bio-pharm Technology Co., Ltd. (Shanghai, China) using the Majorbio Cloud Platform (www.majorbio.com). The thresholds for determining differentially expressed genes were as follows: *p* adjust < 0.05, |Fold change| > 2.

### 2.7. Network Pharmacology Analysis

Network pharmacology analysis was performed according to the methods described in previous studies [13], with minor modifications. Putative targets of Iso-AS-II were retrieved from the TCMSP (http://tcmspw.com/tcmsp.php (accessed on 1 February 2026) and SwissTargetPrediction (http://www.swisstargetprediction.ch/ (accessed on 8 February 2026) databases, standardized using UniProt (https://www.uniprot.org/), and filtered to remove non-human genes. Atherosclerosis-related targets were collected from the GeneCards (https://www.genecards.org/), OMIM (https://omim.org/), and DisGeNET (https://www.disgenet.org/) databases using “atherosclerosis” as the keyword, deduplicated, and corrected with UniProt. Overlapping targets between Iso-AS-II and atherosclerosis were identified using the Venny 2.1.0 online tool (https://bioinfogp.cnb.csic.es/tools/venny/ (accessed on 15 February 2026). The common targets were submitted to the STRING database (https://string-db.org/) to construct a protein–protein interaction (PPI) network, which was then visualized using Cytoscape v3.10.4 (https://cytoscape.org/). Gene Ontology (GO) enrichment and Kyoto Encyclopedia of Genes and Genomes (KEGG) pathway analyses were performed using the DAVID 6.8 database (https://david.ncifcrf.gov/). The top 10 GO terms and top 30 KEGG pathways (with adjusted *p*-value < 0.05) were selected for further analysis.

### 2.8. Foam-Cell Model

RAW 264.7 macrophages (ATCC, Manassas, VA, USA) were cultured in DMEM with 10% FBS, 1% penicillin/streptomycin at 37 °C in 5% CO_2_. Logarithmic-phase cells were seeded, precultured for 12 h, and divided into four groups: control (Con), model (Model), low-dose Iso-AS-II (Iso-AS-II-Low), and high-dose Iso-AS-II (Iso-AS-II-High). The Con group was maintained in complete medium without oxidized low-density lipoprotein (ox-LDL), while the other three groups were treated with 50 μg/mL ox-LDL (#IO1300, Solarbio, Beijing, China) for 24 h to induce foam cell formation [14]. The Low and High groups received 20 and 40 μM Iso-AS-II, respectively, and the Con and Model groups received vehicle alone (*n* = 6 independent experiments). After 24 h, the medium was removed, and cells were washed twice with cold PBS.

### 2.9. Enzyme-Linked Immunosorbent Assay (ELISA)

After the 24 h treatment, cells were collected, washed, and lysed with RIPA buffer. Lysates were centrifuged at 12,000 rpm for 10 min at 4 °C, and the supernatants were collected. Levels of IL-1β, IL-6, and TNF-α in the supernatants were measured using commercial ELISA kits according to the manufacturer’s instructions (Shanghai Enzyme-linked Biotechnology, Shanghai, China).

### 2.10. Real-Time Quantitative PCR

After 24 h of treatment, the culture medium was removed, and cells were washed twice with PBS. Total RNA was isolated from cells using an RNA extraction kit (#RC112, Vazyme Biotech, Nanjing, China), reverse-transcribed into cDNA using a reverse transcription kit (#R333, Vazyme Biotech), and subjected to qPCR with ChamQ Blue Universal SYBR qPCR Master Mix (#Q312, Vazyme Biotech). mRNA levels were normalized to β-actin and quantified using the 2^−ΔΔCt^ method. The primer sequences used in this study are listed in Table 1.

### 2.11. Statistical Analysis

Statistical analysis was conducted using GraphPad Prism 9 (GraphPad Software, San Diego, CA, USA). All results were expressed as mean ± SD. Comparisons among multiple groups were performed using one-way ANOVA with Tukey’s post hoc test. Significance levels were set at * *p* < 0.05, ** *p* < 0.01, *** *p* < 0.001, and **** *p* < 0.0001.

## 3. Results

### 3.1. Iso-AS-II Attenuates Atherosclerosis and Dyslipidemia in ApoE^-/-^ Mice

To evaluate the anti-atherosclerotic effects of Iso-AS-II, ApoE^-/-^ mice were fed a normal chow diet or a WD for 12 weeks, with WD-fed mice receiving either vehicle or Iso-AS-II. As shown in Figure 1A,B, body weight increased progressively across all groups, with no significant differences in either the growth trajectory or cumulative weight gain (Figure 1A,B). Oil Red O staining revealed minimal aortic lipid deposition in the NCD group, whereas WD feeding induced extensive lipid-rich lesions, particularly in the aortic arch and around major branch ostia (Figure 1C). Iso-AS-II treatment visibly reduced the extent of Oil Red O-positive areas. H&E staining of the aortic root showed pronounced atherosclerotic lesion formation in WD-fed mice. Quantitative analysis revealed that the relative lesion area was markedly increased in the WD group compared with the NCD group (*p* < 0.0001), whereas Iso-AS-II treatment significantly reduced the lesion area relative to WD feeding alone (*p* < 0.05) (Figure 1D). Masson staining further showed extensive collagen within aortic root lesions following WD feeding. The collagen-positive area was significantly greater in the WD group than in the NCD group (*p* < 0.001), while Iso-AS-II significantly decreased plaque collagen deposition compared with the WD group (*p* < 0.05) (Figure 1E).

WD feeding significantly increased serum TC, TG, and LDL-C compared with NCD feeding (*p* < 0.01–0.0001) (Figure 1F–H), suggesting WD induced lipid metabolism disorders. Iso-AS-II treatment significantly attenuated these increases (*p* < 0.01–0.0001). WD feeding also increased serum HDL-C relative to the NCD group (*p* < 0.01), whereas Iso-AS-II markedly reduced HDL-C compared with the WD group (*p* < 0.0001) (Figure 1I). In addition, WD feeding significantly elevated serum ALT and AST levels compared with NCD feeding (*p* < 0.01–0.001), both of which were significantly reduced by Iso-AS-II treatment (*p* < 0.01–0.001) (Figure 1J,K).

Collectively, these results suggest that Iso-AS-II effectively attenuates WD-induced atherosclerosis in ApoE^-/-^ mice, as evidenced by reduced aortic lipid deposition, decreased aortic root lesion area, alleviated plaque collagen accumulation, improved serum lipid profiles, and attenuated hepatic injury markers.

### 3.2. Iso-AS-II Suppresses Systemic Inflammatory Cytokines in ApoE^-/-^ Mice

To determine whether the anti-atherosclerotic effects of Iso-AS-II were associated with attenuation of systemic inflammation, serum cytokine concentrations were analyzed using a Luminex multiplex assay. As shown in Figure 2, compared with the NCD group, WD feeding markedly increased serum TNF-α, IL-6, M-CSF, MCP-1 and IL-12 levels (*p* < 0.01–0.0001), suggesting WD induced inflammatory injury. Compared with the WD group, the levels of systemic inflammatory cytokines including TNF-α, IL-6, M-CSF, MCP-1 and IL-12 were significantly suppressed (*p* < 0.05–0.0001) after Iso-AS-II treatment. Notably, no significant differences were observed in serum IL-1β, IL-4, or IL-10 among the three groups (Figure 2B,C,G). These results indicate that Iso-AS-II suppresses WD-induced systemic inflammation in ApoE^-/-^ mice.

### 3.3. Iso-AS-II Modulates Plaque Cellular Composition in ApoE^-/-^ Mice

Immunofluorescence staining was performed to evaluate macrophage accumulation and vascular smooth muscle cell remodeling in the aortic root. F4/80-positive macrophages were rarely detected in the NCD group, whereas WD feeding induced extensive macrophage accumulation within the vascular wall and atherosclerotic lesions (Figure 3). Quantitative analysis showed that F4/80 fluorescence intensity was markedly increased in the WD group compared with the NCD group (*p* < 0.0001). Iso-AS-II treatment substantially reduced F4/80-positive staining relative to the WD group (*p* < 0.0001). α-SMA staining was used to assess smooth muscle cell-associated vascular remodeling. Compared with the NCD group, WD feeding significantly increased α-SMA fluorescence intensity in aortic root sections (*p* < 0.001, Figure 3). Iso-AS-II treatment markedly reduced α-SMA fluorescence intensity compared with the WD group (*p* < 0.001). These results suggest that Iso-AS-II reduced plaque macrophage accumulation and attenuated vascular smooth muscle cell remodeling in the aortic root of WD-fed ApoE^-/-^ mice.

### 3.4. Transcriptomic Analysis of Aortic Tissues

To characterize the molecular changes associated with the anti-atherosclerotic activity of Iso-AS-II, RNA sequencing was performed on aortic tissues. Principal component analysis (PCA) revealed distinct global transcriptional profiles among the three groups (Figure 4A). Samples from the WD group were clearly separated from those in the NCD group, whereas Iso-AS-II-treated samples formed a relatively compact cluster and were positioned closer to the NCD group than to the WD group. The Venn diagram showed that 13,498 genes were commonly expressed across all three groups, with 483, 425, and 257 genes uniquely detected in the WD, NCD, and Iso-AS-II groups, respectively (Figure 4B). Hierarchical clustering of differentially expressed genes between the Iso-AS-II and WD groups showed marked differences in gene expression patterns (Figure 4C). The Iso-AS-II and NCD profiles clustered more closely with each other than with the WD profile. Volcano plot analysis identified 1033 differentially expressed genes between the Iso-AS-II and WD groups, with 210 genes upregulated and 823 downregulated by Iso-AS-II (Figure 4D).

The differentially expressed genes between the Iso-AS-II and WD groups were subjected to GO and KEGG enrichment analyses. GO and KEGG annotation showed that Iso-AS-II-responsive genes were broadly distributed across multiple functional categories (Figure 5A,B). GO enrichment analysis of the genes downregulated by Iso-AS-II identified “intracellular membrane-bounded organelle”, “membrane-bounded organelles” and “localization” (Figure 5C). KEGG enrichment analysis of the downregulated genes revealed significant enrichment of “oxidative phosphorylation”, “glycolysis/gluconeogenesis”, “Gap junction”, “insulin secretion”, “phagosome” and “proteasome” (Figure 5D). These results showed that Iso-AS-II downregulated pathways related to energy metabolism and immune regulation, which may be associated with lipid metabolism and inflammatory responses, paralleling the reduced serum lipids, plaque burden, and systemic inflammation observed in WD-fed mice.

### 3.5. Network Pharmacology Analysis Results

To explore the molecular basis of the anti-atherosclerotic activity of Iso-AS-II, network pharmacology analysis was performed based on its chemical structure (Figure 6A). A total of 182 putative Iso-AS-II-associated targets and 1454 atherosclerosis-related targets were retrieved from public databases. Intersection analysis identified 55 common targets (Figure 6B). These overlapping targets were submitted for PPI analysis, generating a densely connected interaction network (Figure 6C). Topological analysis further identified a core target network in which *AKT1*, *PIK3*, *PPARG*, and *STAT3* occupied highly connected positions (Figure 6D). GO enrichment analysis (Figure 6E) showed that the common targets were mainly associated with biological processes including “regulation of inflammatory responses”, “wound healing” and “muscle cell proliferation”; cellular components such as “cytoplasmic vesicle lumen” and “secretory granule lumen”; and molecular functions including “nuclear receptor activity” and “ligand-activated transcription factor activity”, etc. KEGG pathway enrichment analysis (Figure 6F) identified the “lipid and atherosclerosis” pathway as the most significantly enriched pathway, with the largest number of associated targets. Other significantly enriched pathways included the “AGE-RAGE signaling pathway in diabetic complications”, “C-type lectin receptor signaling”, and “arachidonic acid metabolism”. Collectively, the network pharmacology analysis pointed to a potential role of Iso-AS-II in inflammatory regulation and lipid metabolism.

### 3.6. Iso-AS-II Inhibits Foam Cell Formation

To further validate the anti-inflammatory and cholesterol-modulating effects of Iso-AS-II suggested by the in vivo and network pharmacology results, we employed an ox-LDL-induced RAW 264.7 macrophage foam cell model. Oil Red O staining (Figure 7A) revealed minimal neutral lipid accumulation in untreated macrophages, whereas ox-LDL exposure (50 μg/mL) induced abundant intracellular lipid droplets and a typical foam-cell-like phenotype. Iso-AS-II treatment visibly reduced lipid accumulation, with the high-dose group (40 μM) showing a more pronounced effect than the low-dose group (20 μM).

As shown in Figure 7B–G, compared with the control group, ox-LDL markedly increased the expression of cholesterol uptake genes (*Cd36* and *Msr1*), the cholesterol esterification gene (*Acat1*), and the cholesterol efflux genes (*Abca1* and *Abcg1*) (*p* < 0.05–0.001), and the expression of the cholesteryl ester hydrolase gene (*Nceh1*) was inhibited (*p* < 0.0001). Iso-AS-II treatment significantly suppressed the expression of *Cd36* and *Msr1* (*p* < 0.0001), with no significant difference between the low- and high-dose groups (Figure 7B,C). Iso-AS-II treatment significantly upregulated the expression of *Abca1* and *Abcg1* (*p* < 0.01–0.0001), with the high-dose treatment producing the greatest increase (Figure 7D,E). A similar pattern was observed for *Acat1*, with high-dose Iso-AS-II exerting a stronger inhibitory effect (*p* < 0.001). In parallel, Iso-AS-II treatment significantly upregulated the expression of *Nceh1* (*p* < 0.05–0.001), also in a dose-dependent manner. Ox-LDL-induced lipid accumulation was also accompanied by a marked inflammatory response. The concentrations of IL-1β, IL-6, and TNF-α were significantly elevated in the model group compared with the control group (all *p* < 0.0001) (Figure 7H–J). Both concentrations of Iso-AS-II significantly reduced the levels of these cytokines (*p* < 0.01–0.001), with the high-dose treatment generally restoring them toward or below basal levels. Together, these results suggest that Iso-AS-II attenuated macrophage cholesterol accumulation and foam-cell-associated inflammation, with the high dose generally producing greater effects.

## 4. Discussion

Atherosclerosis remains the leading cause of cardiovascular morbidity and mortality worldwide, driven by arterial lipid accumulation and unresolved inflammation [15]. Given that considerable residual risk persists despite current lipid-lowering therapy, natural products combining lipid-modulating and anti-inflammatory activities are increasingly sought [16]. Saponins from *A. membranaceus* have been investigated for their cardiovascular benefits, but almost exclusively as astragaloside IV [8]. Iso-AS-II, a structurally related cycloartane saponin, has so far been reported mainly to exert anti-inflammatory and hepatoprotective effects [9,10]. To the best of our knowledge, this is the first study to report that Iso-AS-II attenuates atherosclerosis.

The ApoE^-/-^ mouse is one of the most widely used animal models of atherosclerosis. In the well-established WD-fed ApoE^-/-^ mouse model [17], Iso-AS-II reduced aortic lesion area and plaque collagen deposition in parallel with marked decreases in serum TC, TG, and LDL-C, indicating improved systemic lipid homeostasis. This lipid-modulating profile is consistent with the anti-atherosclerotic activity reported for astragaloside IV [18,19] and supports the potential of Iso-AS-II as a natural lipid-lowering agent. Iso-AS-II also lowered serum HDL-C in WD-fed mice. HDL-C is abnormally elevated in this model and does not reflect HDL function, which predicts cardiovascular protection better than HDL-C concentration does [20]; the significance of this change therefore remains unclear and merits investigation. Finally, reduced serum ALT and AST point to alleviation of WD-induced hepatic injury, in line with the hepatoprotective activity of Iso-AS-II in cholestatic liver disease [10]. Inflammation is a central driver of atherosclerosis and an attractive therapeutic target [16]. Iso-AS-II significantly lowered serum TNF-α, IL-6, MCP-1, M-CSF, and IL-12 in vivo and reduced IL-1β, IL-6 and TNF-α levels in ox-LDL-stimulated macrophages. MCP-1 and M-CSF are key regulators of monocyte recruitment and plaque macrophage expansion [21], and their suppression fits the reduced F4/80-positive macrophage accumulation in the aortic root. Together with the reported inhibition of nitric oxide production by Iso-AS-II in macrophages [9] and the anti-inflammatory activity of other Astragalus saponins [8], these results highlight the anti-inflammatory potential of Iso-AS-II. Because cholesterol metabolism and inflammation are closely intertwined in atherogenesis [22], its lipid-modulating and anti-inflammatory actions may act synergistically.

Aortic RNA sequencing revealed that Iso-AS-II downregulated pathways related to “oxidative phosphorylation”, “glycolysis/gluconeogenesis” and “phagosome”, processes closely linked to macrophage metabolic reprogramming in atherosclerosis [23]. Macrophage inflammatory activation and foam-cell formation are accompanied by profound metabolic reprogramming [24]; these changes suggest that Iso-AS-II dampens the energy-demanding metabolic programs that sustain plaque inflammation. Concurrently, network pharmacology identified a set of inflammation-related targets (*AKT1*, *PIK3*, *STAT3*, etc.) as core targets of Iso-AS-II, with KEGG enrichment pinpointing “lipid and atherosclerosis” as the most significantly enriched pathway. Together, these in silico and transcriptomic data suggest that lipid metabolism and inflammatory regulation are involved, and support the hypothesis that Iso-AS-II reduces atherosclerosis in part through modulation of macrophage cholesterol homeostasis and inflammatory responses. Therefore, we further employed an ox-LDL-induced foam cell model to investigate the effects of Iso-AS-II on anti-inflammatory responses and cholesterol metabolism regulation.

Foam-cell formation is a hallmark of atherogenesis [25]. The mechanisms underlying macrophage foam cell formation involve a complex interplay of cholesterol uptake, esterification, and efflux. Cholesterol uptake by macrophages and other cells occurs through multiple transporters, among which CD36 and MSR1 account for 75–90% of modified lipoprotein internalization [26]. ACAT1 mediates cholesterol esterification for intracellular storage, whereas NCEH1 hydrolyzes cholesteryl esters to free cholesterol for efflux [26]. Maintaining a balance between these processes-rather than simply promoting or inhibiting -is crucial for foam cell homeostasis, with the goal of facilitating cholesterol efflux via ABCA1/ABCG1. ABCA1 and ABCG1 are key transporters mediating cholesterol efflux from macrophages to extracellular acceptors, contributing 60–70% of efflux under lipid-overload conditions [26]. In ox-LDL-stimulated macrophages, Iso-AS-II downregulated the expression of scavenger genes *Cd36* and *Msr1* that mediate modified lipoprotein uptake and upregulated the expression of cholesterol efflux genes *Abca1* and *Abcg1*. The expression levels of *Acat1* and *Nceh1* were downregulated and upregulated, respectively. These findings suggest that Iso-AS-II shifts the cholesterol balance in macrophages from storage toward efflux and hydrolysis, downregulating uptake and esterification while upregulating hydrolysis and efflux, ultimately attenuating foam cell formation.

Several limitations should be acknowledged. (i) Only a single dose of Iso-AS-II (5 mg/kg, i.p.) was tested, without dose–response assessment or a positive control, and the intraperitoneal route bypasses the gastrointestinal tract. Given that *A. membranaceus* saponins undergo extensive metabolism after oral administration [27], oral bioavailability and efficacy require dedicated study. (ii) Since hepatic steatosis and targets related to hepatic lipogenesis and lipoprotein metabolism were not evaluated, the mechanisms underlying the observed reductions in triglycerides and cholesterol remain unclear. Given the liver’s central role in lipid homeostasis, these effects should be interpreted as descriptive/exploratory, and future studies should directly assess hepatic lipid content and related molecular pathways. (iii) The transcriptomic and network pharmacology findings were not confirmed at the protein level, and the in vivo contribution of macrophage cholesterol handling to atherogenesis was not directly demonstrated. Future studies with multiple doses, oral formulations, and protein-level validation, as well as larger, better-designed studies to independently replicate and confirm the present findings, are needed to establish the therapeutic potential of Iso-AS-II for atherosclerosis. (iv) To our knowledge, although the total astragaloside content of *A. membranaceus* is well characterized [28], the Iso-AS-II content in different *A. membranaceus* sources and the corresponding human dose have not been reported, limiting direct comparison with the animal dose; future studies should therefore include Iso-AS-II-specific chemical characterization and pharmacokinetic or clinical dose data.

## 5. Conclusions

In conclusion, this exploratory study provides preliminary evidence that Iso-AS-II may attenuate atherosclerotic lesion burden in WD-fed ApoE^-/-^ mice and ox-LDL-stimulated macrophages. The compound improves serum lipid profiles, suppresses systemic inflammation, and attenuates aortic plaque formation. In macrophages, Iso-AS-II downregulates cholesterol uptake and esterification while upregulating hydrolysis and efflux, correlating with reduced foam cell formation. These findings suggest that Iso-AS-II may be a promising natural candidate for further investigation in atherosclerosis.

## Figures and Tables

**Figure 1:**
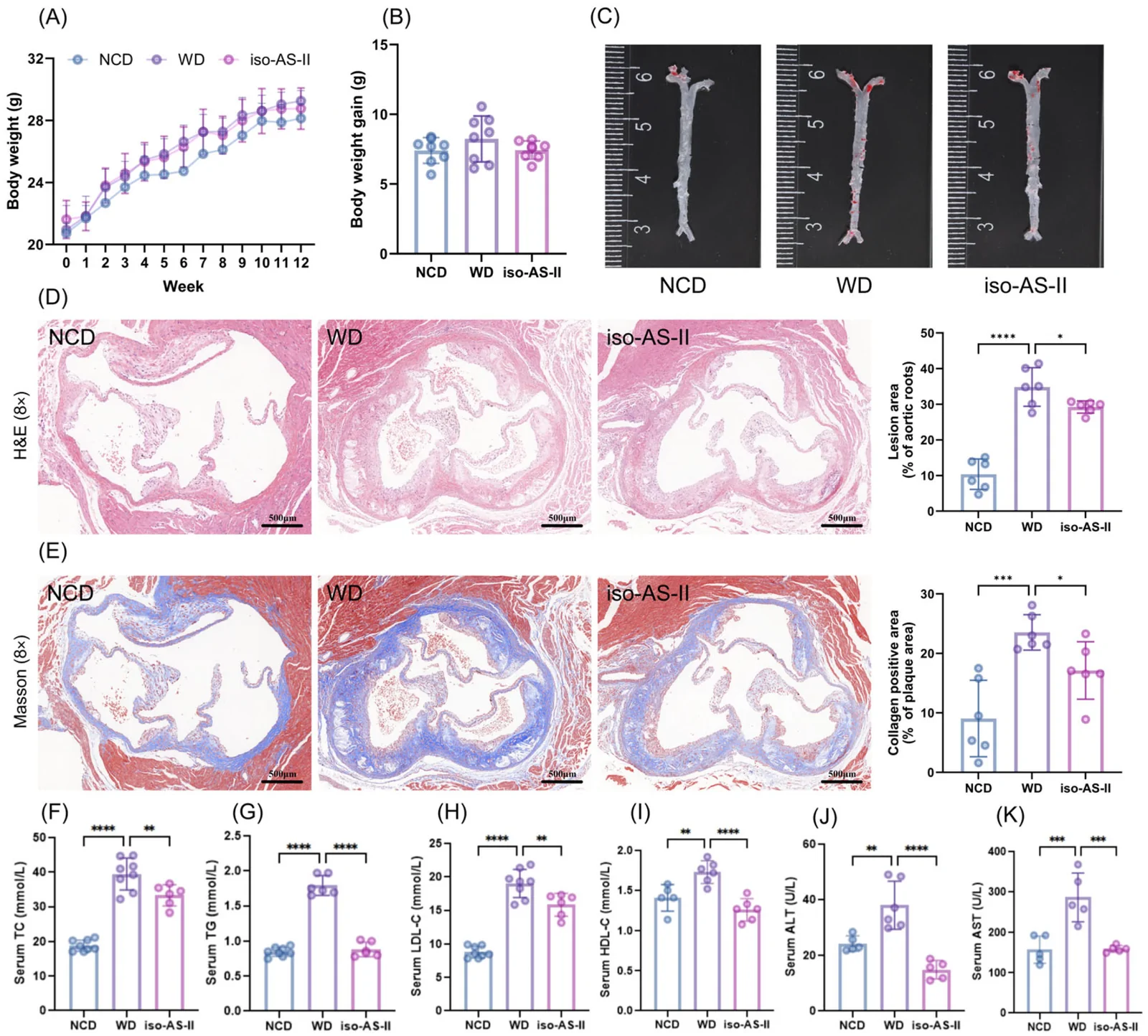
Iso-AS-II attenuated WD-induced atherosclerosis and dyslipidemia in ApoE^-/-^ mice. (**A**,**B**) Body weight changes and body weight gain, (**C**) Oil Red O staining of mouse aortic arch, (**D**) H&E staining of mouse aortic root, (**E**) Masson staining of mouse aortic root, (**F**–**I**) Serum levels of TC, TG, LDL-C and HDL-C, (**J**,**K**) Serum levels of ALT and AST. (*n* = 5–8 mice per group, * *p* < 0.05, ** *p* < 0.01, *** *p* < 0.001, and **** *p* < 0.0001).

**Figure 2:**
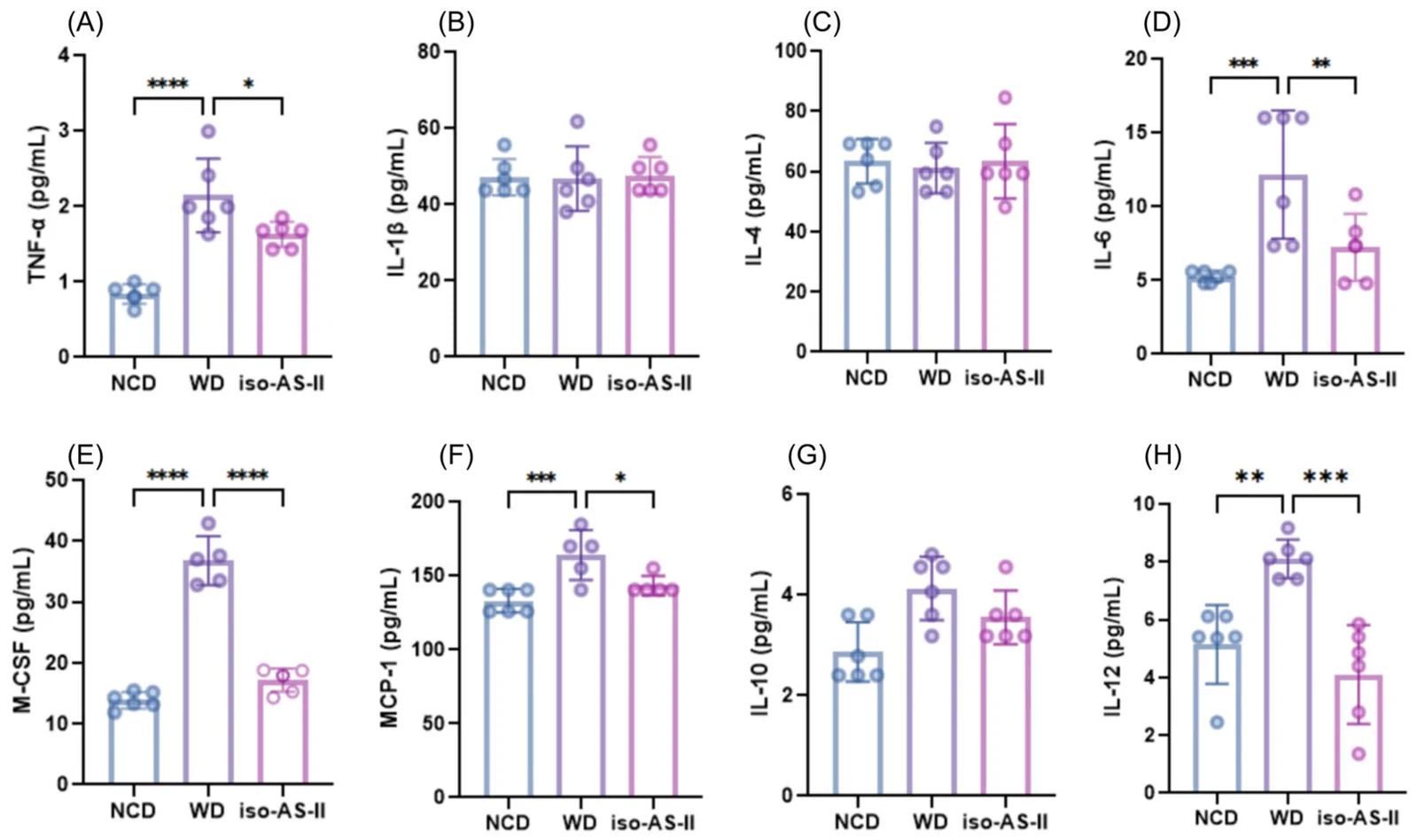
Iso-AS-II suppresses WD-induced systemic inflammation in ApoE^-/-^ mice. (**A**–**H**) Serum cytokine levels of TNF-α, IL-1β, IL-4, IL-6, M-CSF, MCP-1, IL-10, and IL-12. (*n* = 5–6 mice per group, * *p* < 0.05, ** *p* < 0.01, *** *p* < 0.001, and **** *p* < 0.0001).

**Figure 3:**
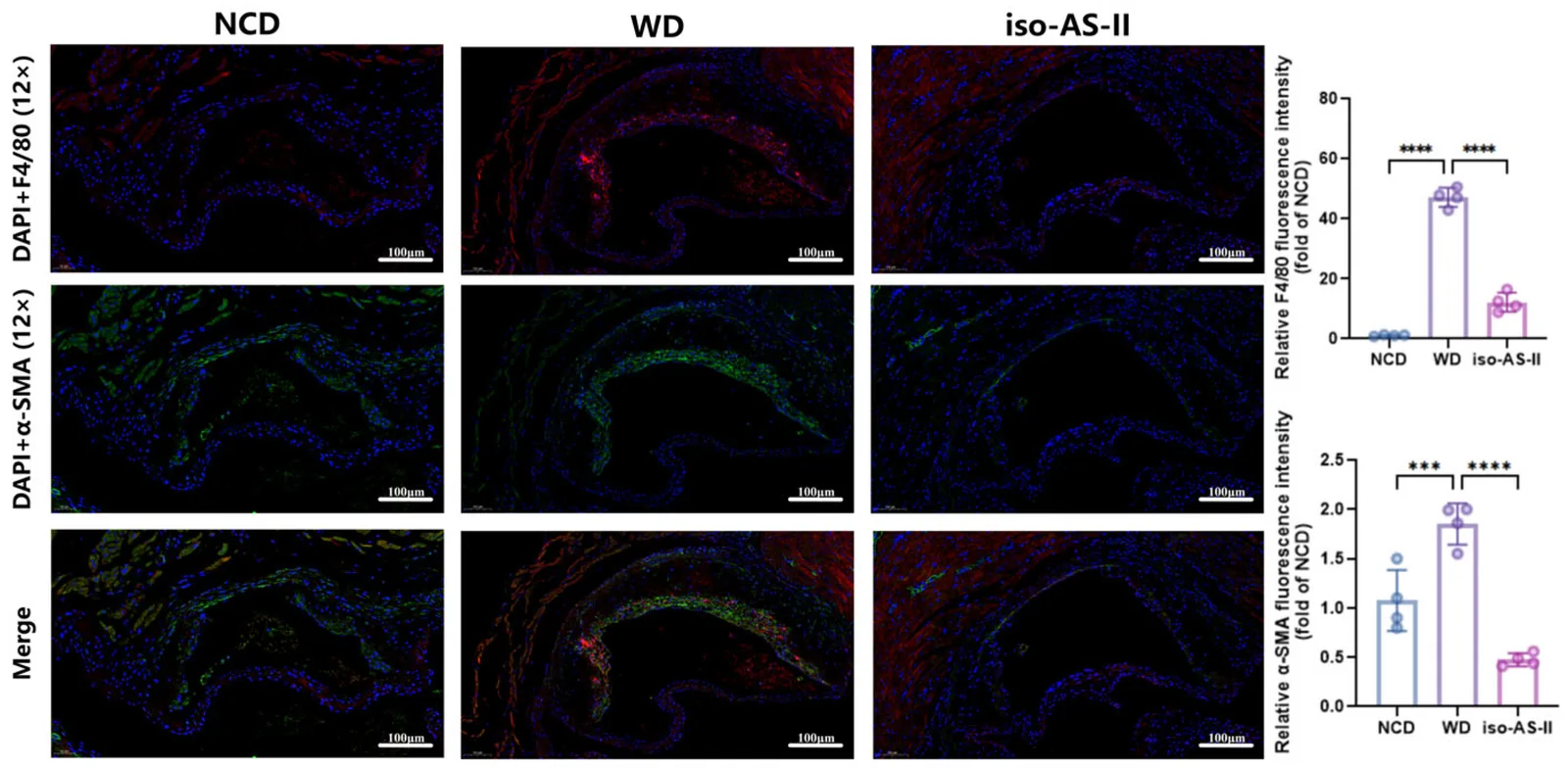
Immunofluorescence staining for F4/80 and α-SMA in the aortic root of mice. Red indicates F4/80; green indicates α-SMA; blue indicates DAPI-stained nuclei. (*n* = 4 mice per group, *** *p* < 0.001, and **** *p* < 0.0001).

**Figure 4:**
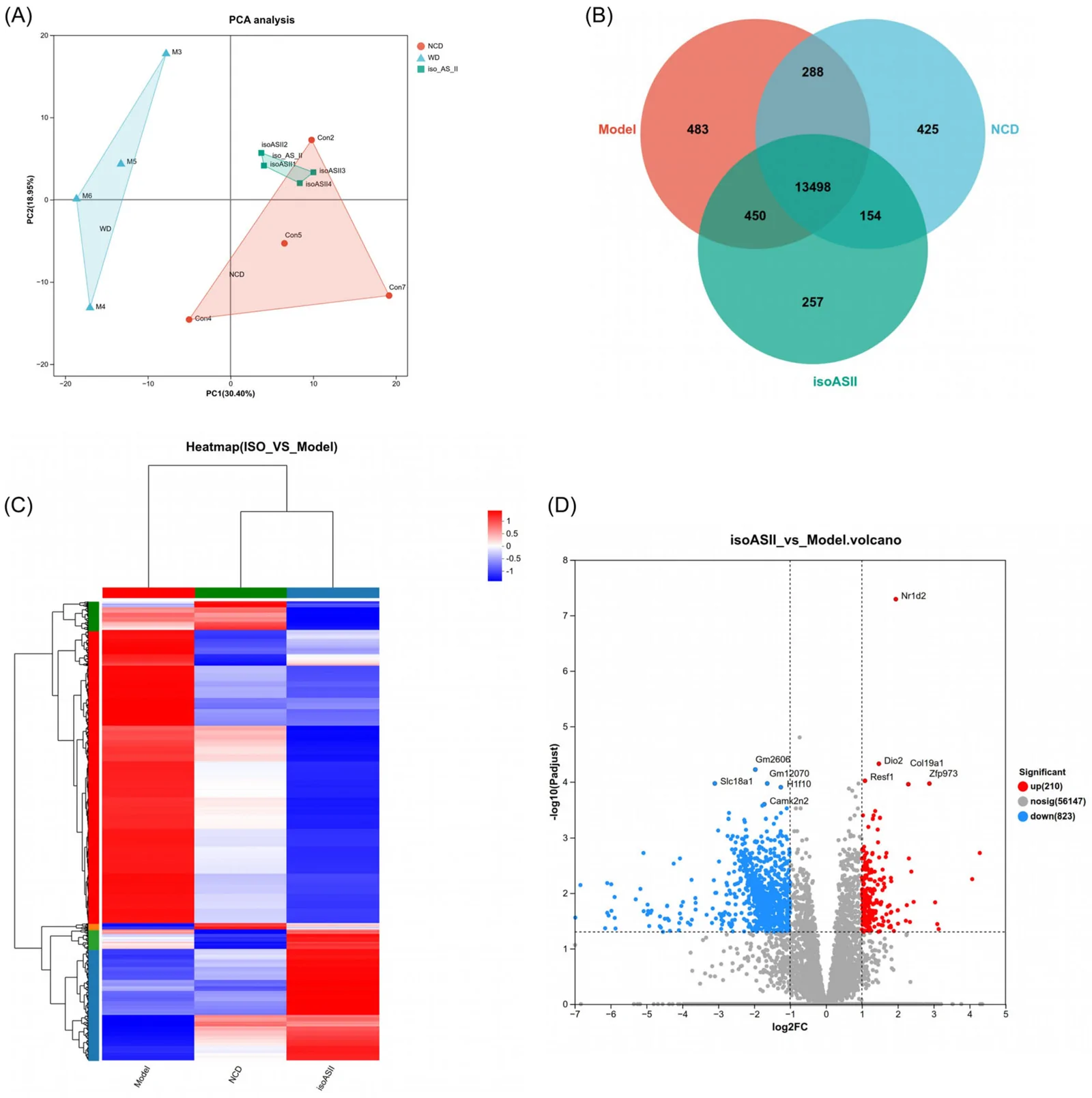
Transcriptome profiles in aortas of different groups. (**A**) PCA analysis, (**B**) Venn analysis of DEGs, (**C**) Hierarchical clustering, (**D**) Volcano diagrams (*n* = 4 mice per group). Original figures are provided in the Supplementary Material (PowerPoint file) for better readability.

**Figure 5:**
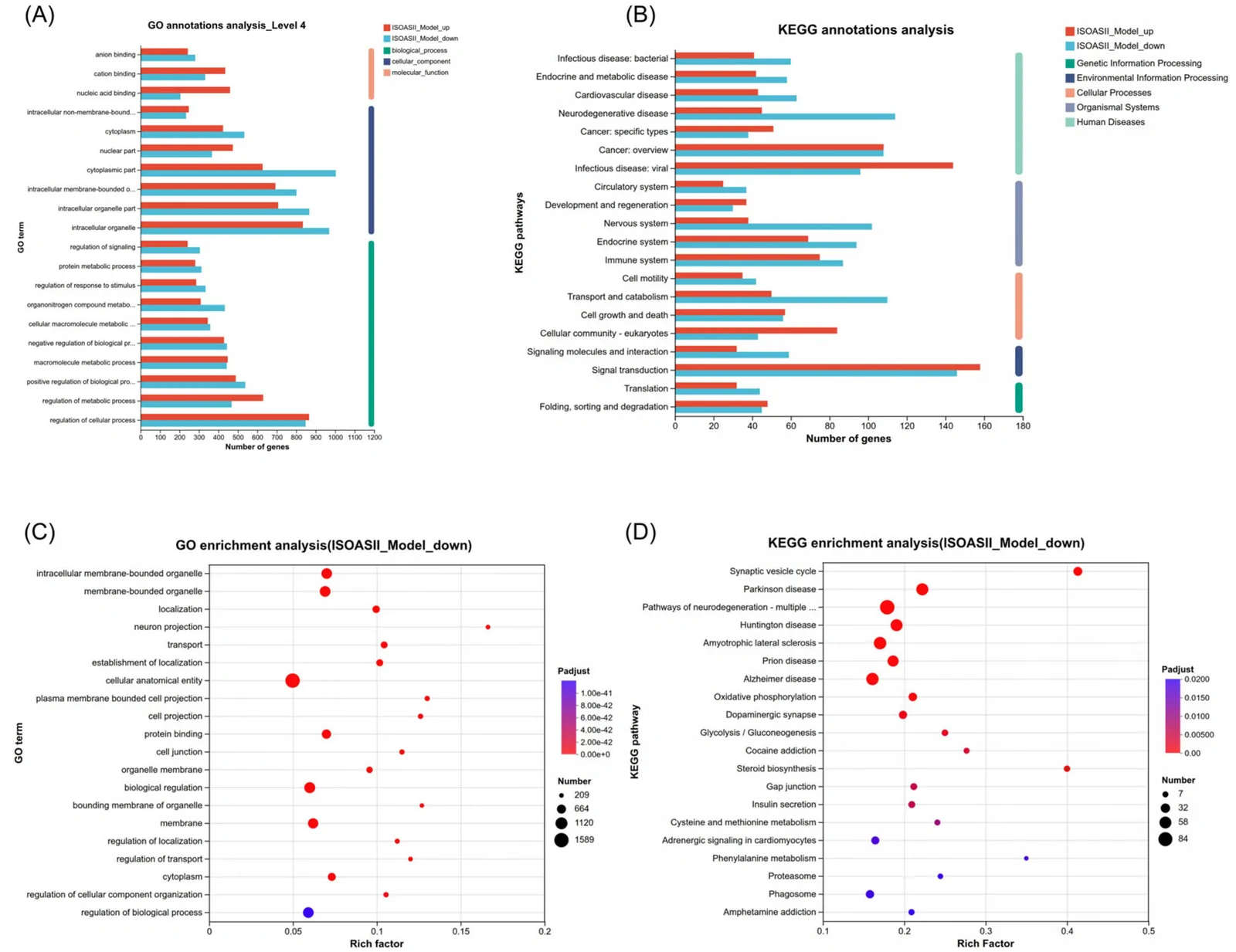
GO and KEGG analysis. (**A**,**B**) GO and KEGG annotation, (**C**,**D**) GO and KEGG enrichment analysis (*n* = 4 mice per group). Original figures are provided in the Supplementary Material (PowerPoint file) for better readability.

**Figure 6:**
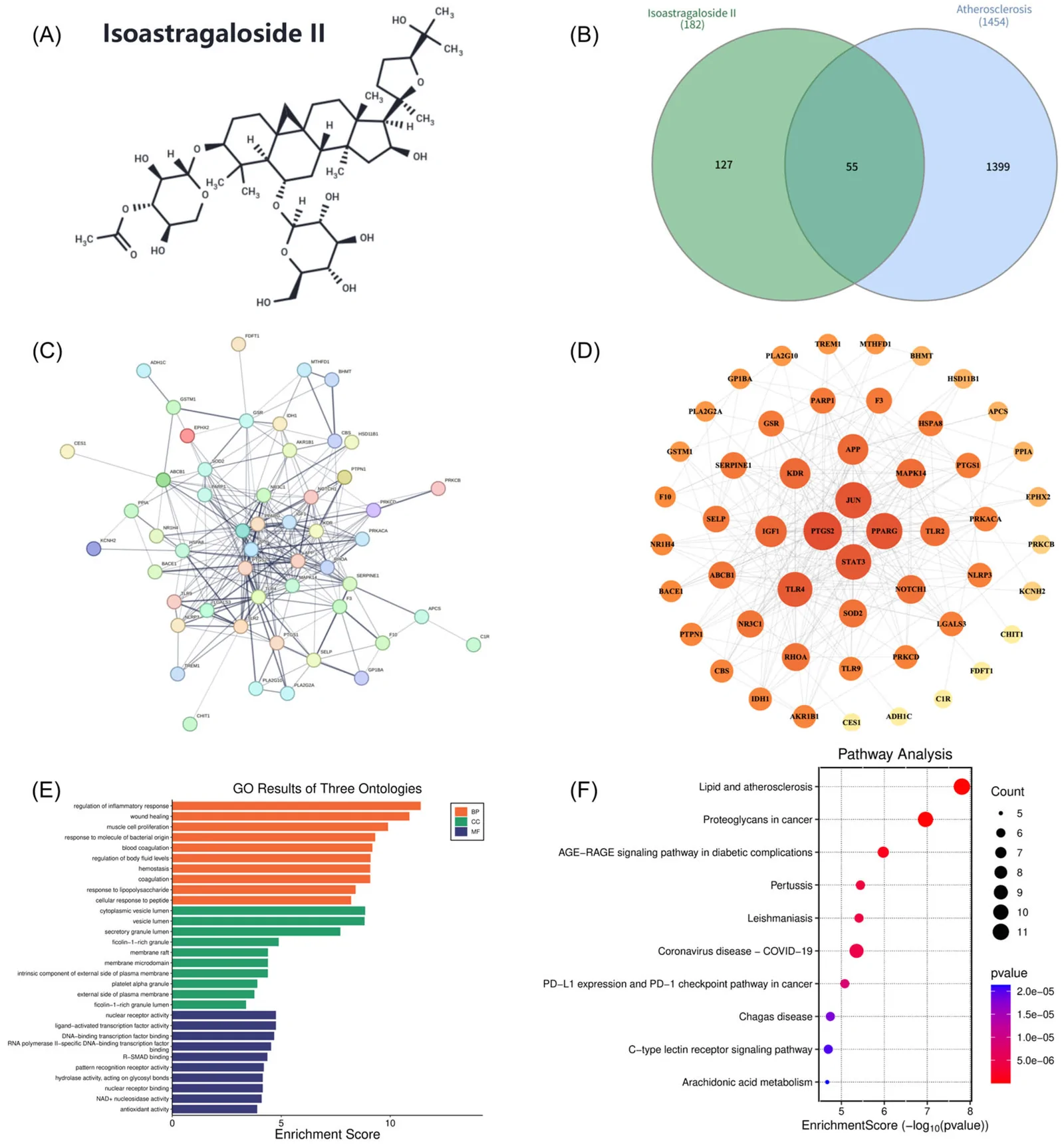
Network pharmacology analysis of the potential anti-atherosclerotic mechanisms of Iso-AS-II. (**A**) Chemical structure of Iso-AS-II. (**B**) Venn diagram of overlapping targets between Iso-AS-II and atherosclerosis. (**C**) PPI network of common targets. (**D**) Core target network identified by topological analysis. (**E**) GO enrichment. (**F**) KEGG pathway enrichment. Original figures are provided in the Supplementary Material (PowerPoint file) for better readability.

**Figure 7:**
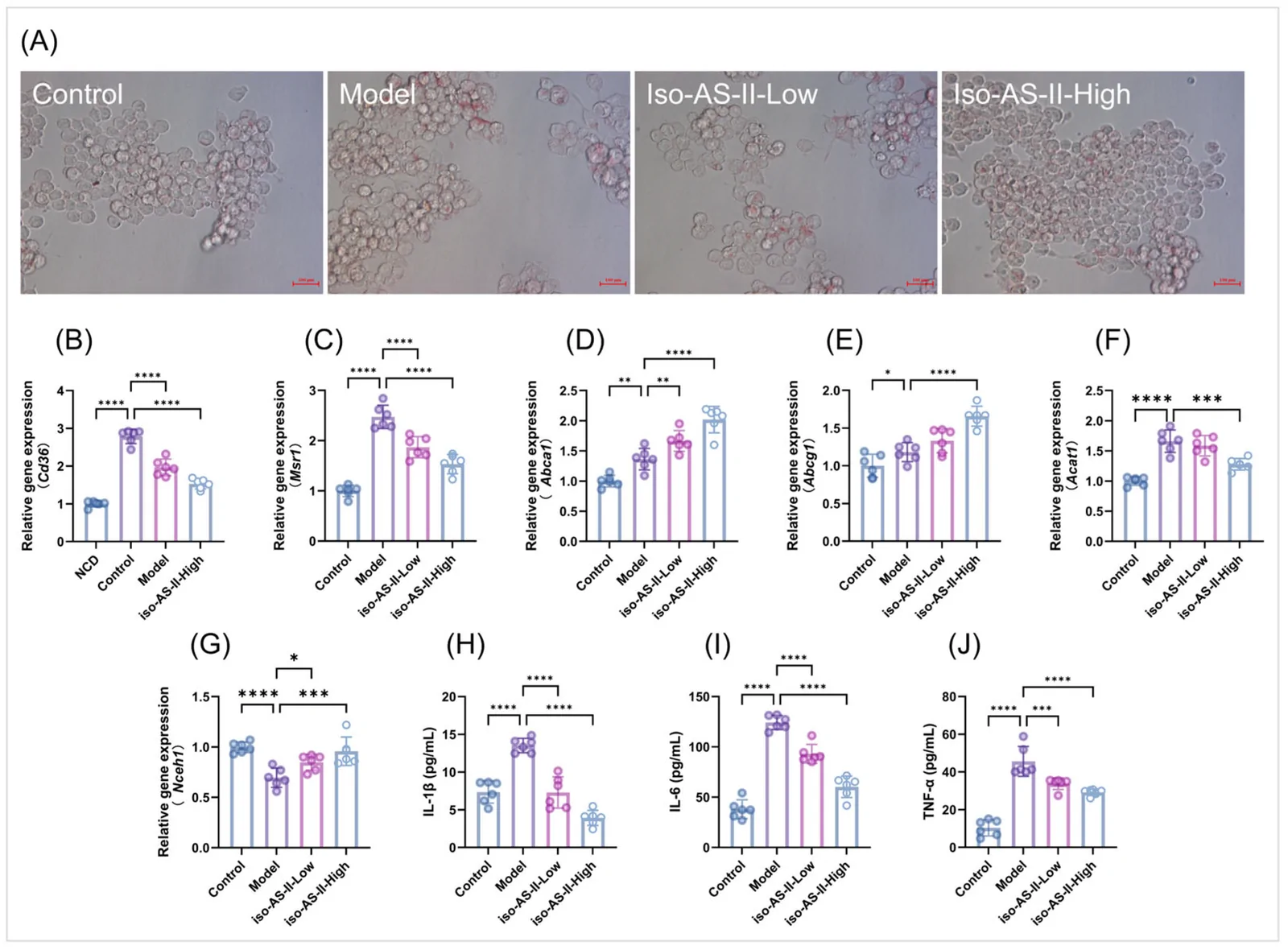
Effects of Iso-AS-II on ox-LDL-induced foam cell formation in RAW 264.7 macrophages. (**A**) Oil Red O staining of intracellular lipid accumulation (the scale is 100 μm), (**B**–**G**) Relative mRNA expression of genes *Cd36*, *Msr1*, *Abca1*, *Abcg1, Acat1* and *Nceh1*, (**H**–**J**) Concentrations of inflammatory cytokines IL-1β, IL-6, and TNF-α in cell lysates. (*n* = 6 independent experiments, * *p* < 0.05, ** *p* < 0.01, *** *p* < 0.001, and **** *p* < 0.0001).

**Table 1:** The primer sequences for qPCR.

Gene	Forward Primer (5′→3′)	Reverse Primer (5′→3′)
*Acat1*	CAGGAAGTAAGATGCCTGGAAC	TTCACCCCCTTGGATGACATT
*Nceh1*	TTGAATACAGGCTAGTCCCACA	CAACGTAGGTAAACTGTTGTCCC
*Cd36*	ATGGGCTGTGATCGGAACTG	GTCTTCCCAATAAGCATGTCTCC
*Sr-a (Msr1)*	TGAACGAGAGGATGCTGACTG	TGTCATTGAACGTGCGTCAAA
*Abca1*	AAAACCGCAGACATCCTTCAG	CATACCGAAACTCGTTCACCC
*Abcg1*	CCGATGTCCCCTTTCAGATCA	GAGCAGCGAACAGCACAAAA
*β-actin*	GGCTGTATTCCCCTCCATCG	CCAGTTGGTAACAATGCCATGT

## Data Availability

The original contributions presented in this study are included in the article. Further inquiries can be directed to the corresponding author.

## Supplementary Materials

The following supporting information can be downloaded at: https://www.mdpi.com/article/10.3390/nu18193137/s1, Table S1: The compositions of the NCD and WD diets.
