# Supplementary material for: Intraperitoneal Isoastragaloside II Attenuated Western Diet-Induced Atherosclerosis in Male ApoE-/- Mice with Lipid-Lowering and Anti-Inflammatory Effects
Source: Nutrients. 2026 Sep 23;18(19):3137. doi: 10.3390/nu18193137 (PMC13666752; doi:10.3390/nu18193137)
Supplement: Supplementary file 1 [file nutrients-18-03137-s001.zip › nutrients-4546466-supplementary.pdf]

Supplementary Material

1. Diet composition

Table S1 The compositions of the NCD and WD diets

(NCD: XT102C; WD: XT108C)

A. Nutritional level

Nutrient	XT108C by weight (%)	XT108C by energy (%)	XT102C by weight (%)	XT102C by energy (%)
Protein	22.62	19.97	19.00	20.00
Carbohydrate	45.51	40.18	67.00	70.00
Fat	20.06	39.85	4.00	10.00
Total	-	100.00	-	100.00
kcal/g	4.53	-	3.85	-

B. Composition

Component	XT108C (g)	XT108C (kcal)	XT102C (g)	XT102C (kcal)
Casein	200.00	800.00	200.00	800.00
L-Cystine	3.00	12.00	3.00	12.00
Corn starch	212.00	848.00	386.15	1544.60
Maltodextrin	71.00	284.00	125.00	500.00
Sucrose	124.41	497.64	200.00	800.00
Cellulose	50.00	0.00	50.00	0.00
Soybean oil	25.00	225.00	25.00	225.00
Cocoa butter	155.00	1395.00	20.00	0.00
Mineral mix S10020	5.00	0.00	5.00	0.00
Dicalcium phosphate	13.00	0.00	13.00	0.00
Calcium carbonate	5.50	0.00	5.50	0.00
Potassium citrate, 1 H2O	16.50	0.00	16.50	0.00
Sodium chloride	2.59	0.00	2.59	0.00
Vitamin mix V10001C	1.00	4.00	1.00	4.00
Choline bitartrate	2.00	0.00	2.00	0.00
Cholesterol	11.25	0.00	0.00	0.00
FD&C Yellow Dye #5	0.05	0.00	0.10	0.00
FD&C Red Dye #40	0.00	0.00	0.00	0.00
FD&C Blue Dye #1	0.05	0.00	0.00	0.00
**Total**	**897.35**	**4065.64**	**1054.84**	**4065.60**

2. Number of animals analysed per assay

Twenty-four male ApoE$^{-/-}$ mice were divided into three groups (n = 8 per group), and no mice died during the study. At harvest, each mouse aorta was divided into two portions: one fixed for histological analysis and the other frozen for RNA extraction. Because en face preparation of the aorta had a low success rate, only one aorta with satisfactory en face preparation was obtained per

group; the Oil Red O staining results are therefore presented as representative images rather than as quantitative data. As the different analyses required different amounts of serum and tissue, and not all samples were processed successfully, the number of samples differed among assays. Specifically:

(1) Oil Red O staining (Fig. 1C): one representative specimen per group;

(2) H&E and Masson staining (Fig. 1D,E): 6 mice per group;

(3) Serum biochemical analysis (Fig. 1F-K): 5-8 mice per group, with a small number of samples not measured because of unsuccessful processing;

(4) Luminex multiplex cytokine assay (Fig. 2): 5-6 mice per group, with a small number of samples not measured because of unsuccessful processing;

(5) Immunofluorescence staining (Fig. 3): 4 mice per group;

(6) RNA sequencing (Fig. 4-5): 4 mice per group.

Outliers. No data points were excluded as outliers; therefore, no outlier removal procedure was applied
